# What every intensivist should know about light sedation for mechanically ventilated patients

**DOI:** 10.5935/0103-507X.20210069

**Published:** 2021

**Authors:** Lilian Maria Sobreira Tanaka, Rodrigo Bernardo Serafim, Jorge Ibrain Figueira Salluh

**Affiliations:** 1 Critical Care Department, Hospital Copa D’Or - Rio de Janeiro (RJ), Brazil.; 2 Internal Medicine Department, Universidade Federal do Rio de Janeiro, Rio de Janeiro (RJ), Brazil.; 3 Department of Critical Care and Postgraduate Program in Translational Medicine, Instituto D’Or de Pesquisa e Ensino - Rio de Janeiro (RJ), Brazil.

## INTRODUCTION

Pain, agitation and anxiety are frequently experienced by patients requiring intensive care unit (ICU) admission. These events are often associated with tracheal intubation, mechanical ventilation (MV) and bedside procedures^1^. Sedatives and analgesics can be used to minimize distress, ensure comfort, and decrease the work of breathing to achieve better synchrony with the ventilator.^2^ A number of landmark studies have been published in the past decade, improving our understanding about the choice of sedatives and how their use affects the short- and long-term outcomes of critically ill patients.^2^^,^^3)^ One of the key evidence-based concepts that emerged from observational studies and randomized controlled trials was a protocolized light sedation approach, which was included in recent guidelines.^3^ Light sedation is considered the ideal target for most mechanically ventilated patients, where a “calm, comfortable and collaborative” state can ensure synchronous ventilation with minimal pain and anxiety, coupled with cognitive preservation. Potential patient-centered benefits of light sedation also include the possibility of active cognitive and motor stimulation (including early mobilization interventions) as well as improved interaction with the health care team and family members.^4^

### What is the evidence behind light sedation?

Strong evidence demonstrates that oversedation is associated with worse clinical outcomes, and most recently, special attention has been given to the intensity of sedation in the early phase of MV. Studies demonstrate that allowing deep sedation even in the first 48 hours of MV can be detrimental. In a prospective multicenter longitudinal study on sedation practices comprising patients under MV for a period of 24 hours or more, Shehabi et al. demonstrated that early deep sedation was independently associated with longer time to extubation, hospital death and 180-day mortality.^5^ Similarly, an observational prospective multicenter study including 322 patients from 45 Brazilian ICUs showed that deep sedation within the first 48 hours of MV was independently associated with a 2-fold increase in hospital mortality.^6^ As “light sedation” uses fewer drugs and reduces overall resource use, it can be considered a cost-effective intervention in the ICU. Additionally, deep sedation is associated with worse functional and cognitive outcomes, as it decreases the possibility of early mobilization and significantly increases the risk of *delirium*.^7^

Having a deeply sedated, immobilized patient transition to an awake and cooperative patient is an essential part of best practices in the ICU. However, it is not without its challenges. The ICU team must assure adequate control of potential distress and reduction of adverse outcomes using a multidisciplinary approach. Monitoring for pain and agitation is essential not only to the patients’ well-being but also for safety reasons, as an agitated patient may inadvertently remove intravascular devices or the endotracheal tube. Studies using light sedation have found that patients who are more awake and aware can contribution to their pain evaluations through reliable self-report, *delirium* assessments and early rehabilitation.^3^^,^^4^ Light sedation was also associated with reduced ICU length of stay and shorter duration of MV with no increases in anxiety and depression^8^. In studies where long-term follow-ups were reported, there was no sign of increased negative neuropsychological outcomes.^9^

### Who should receive light sedation in the intensive care unit?

The 2018 Pain, Agitation/Sedation, *Delirium*, Immobility and Sleep Disruption (PADIS) guidelines suggested a protocol-based, stepwise assessment for pain control and sedation management in critically ill adults.^3^ Clearly, the emphasis should not be on sedation but rather on multidisciplinary approaches to monitor, prevent and promptly treat pain and agitation while ensuring participation by an awake and aware patient. Light sedation was recommended for most patients to reduce anxiety and stress, to control symptoms of hyperactive *delirium*, and to facilitate invasive procedures and MV.^3^^,^^10^ Additionally, the early comfort using analgesia, minimal sedatives and maximal human care (eCASH)^4^ and the ABCDF-R bundle (R = respiratory-drive-control)^11^^,^^12)^ guidelines emphasize the use of analgesia first with minimal sedation, communication aids, noise reduction to facilitate good sleep, early mobilization, *delirium* monitoring and family involvement as strategies to promote patient-centered care and comfort in the ICU.

Despite no universal definition of light sedation, guidelines considered a Richmond Agitation Sedation Scale (RASS) score of between +1 (slightly restless) and -2 (awake with eye contact to voice) or a Riker Sedation-Agitation Scale (SAS) score of between 4 (calm and cooperative) and 3 (difficult to rouse and obey simple commands) adequate for most patients.^3^^,^^10^ Strategies to achieve light sedation such as daily interrupted sedation, targeted sedation or even no sedation can be used without a clear superiority of one over the other.^10^^,^^13)^ A preference for the use of fast-acting sedative agents may allow dose titration and adjustment to the target level of consciousness.^14^

Propofol or dexmedetomidine is recommended over benzodiazepines in patients requiring continuous sedation to achieve early^3^^,^^5^^,^^15)^ and continuous light sedation^3^^,^^16)^ and to minimize the risk of *delirium*.^15^ In sepsis patients, propofol and dexmedetomidine have been shown to be comparable in terms of clinical outcomes when light sedation was targeted.^17^ Opioids remain a mainstay for pain management in the ICU,^3^^,^^18^ but the use of adjuvant analgesic therapy, such as acetaminophen, clonidine, dexmedetomidine, gabapentin, ketamine, pregabalin, and tramadol, promotes a reduction in pain scores as well as a reduction in opioid consumption, as demonstrated in a recent meta-analysis.^18^ Only a minority of the patients admitted to the ICU have a clear indication for continuous deep sedation: patients with severe respiratory failure, status epilepticus, intracranial hypertension and the need for neuromuscular blockade.^19)^ Patients with these conditions may be underrepresented in studies on analgesia and sedation because they are frequently excluded.^10^^,^^19)^ However, even when deep sedation is needed, it should be considered a transitory strategy, and the use of combinations of sedatives may be used to minimize the use of benzodiazepines.^19^

**Figure 1:**
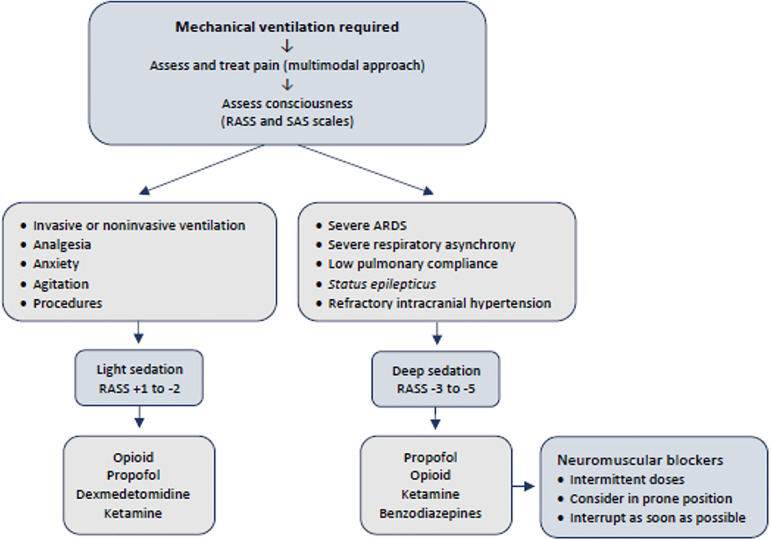
Schematic approach to analgesia and sedation.

A schematic approach to analgesia and sedation is suggested in figure 1.

RASS - Richmond Agitation Sedation Scale; SAS - Riker Sedation-Agitation Scale; ARDS - acute respiratory distress syndrome. Benzodiazepines should be used as the last option for sedation and interrupted as soon as possible.

## CONCLUSION

In conclusion, recent studies demonstrate that the use of light sedation is feasible and safe in most mechanically ventilated patients in the intensive care unit. The shift from a deeply sedated patient to a calm, comfortable and collaborative patient is associated with reduced intensive care unit stay, duration of mechanical ventilation and *delirium* as well as improved survival rates. The use of light sedation is a cost-effective, evidence-based strategy that should be considered the standard of care in the intensive care unit.
